# β-Glucan-Functionalized Nanoparticles Down-Modulate the Proinflammatory Response of Mononuclear Phagocytes Challenged with *Candida albicans*

**DOI:** 10.3390/nano12142475

**Published:** 2022-07-19

**Authors:** Tânia Lima, Stefán B. Gunnarsson, Elisabete Coelho, Dmitry V. Evtuguin, Alexandra Correia, Manuel A. Coimbra, Tommy Cedervall, Manuel Vilanova

**Affiliations:** 1 I3S-Instituto de Investigação e Inovação em Saúde, Universidade do Porto, 4200-135 Porto, Portugal; alexandra.correia@ibmc.up.pt (A.C.); vilanova@icbas.up.pt (M.V.); 2 IBMC-Instituto de Biologia Molecular e Celular, Universidade do Porto, 4200-135 Porto, Portugal; 3 ICBAS-Instituto de Ciências Biomédicas de Abel Salazar, Universidade do Porto, 4050-313 Porto, Portugal; 4 Department of Biochemistry and Structural Biology, Lund University, 221 00 Lund, Sweden; sbragig@gmail.com (S.B.G.); tommy.cedervall@biochemistry.lu.se (T.C.); 5 NanoLund, Center for Nanoscience, Lund University, 221 00 Lund, Sweden; 6 LAQV-REQUIMTE, Departamento de Química, Universidade de Aveiro, 3810-193 Aveiro, Portugal; ecoelho@ua.pt (E.C.); mac@ua.pt (M.A.C.); 7 CICECO, Department of Chemistry, University of Aveiro, 3810-193 Aveiro, Portugal; dmitrye@ua.pt

**Keywords:** *Candida albicans*, β-glucan, nanoparticles, infection, inflammation

## Abstract

Systemic fungal infections are associated with significant morbidity and mortality, and *Candida albicans* is the most common causative agent. Recognition of yeast cells by immune cell surface receptors can trigger phagocytosis of fungal pathogens and a pro-inflammatory response that may contribute to fungal elimination. Nevertheless, the elicited inflammatory response may be deleterious to the host by causing excessive tissue damage. We developed a nanoparticle-based approach to modulate the host deleterious inflammatory consequences of fungal infection by using β1,3-glucan-functionalized polystyrene (β-Glc-PS) nanoparticles. β-Glc-PS nanoparticles decreased the levels of the proinflammatory cytokines TNF-α, IL-6, IL-1β and IL-12p40 detected in in vitro culture supernatants of bone marrow-derived dendritic cells and macrophage challenged with *C. albicans* cells. Moreover, β-Glc-PS nanoparticles impaired the production of reactive oxygen species by bone marrow-derived dendritic cells incubated with *C. albicans*. This immunomodulatory effect was dependent on the nanoparticle size. Overall, β-Glc-PS nanoparticles reduced the proinflammatory response elicited by fungal cells in mononuclear phagocytes, setting the basis for a targeted therapy aimed at protecting the host by lowering the inflammatory cost of infection.

## 1. Introduction

In recent decades, the incidence of human fungal infections has increased, especially in immunocompromised and hospitalized individuals. The fungal infection associated morbidity and mortality are significant, and it is clear that these infections have emerged as important public health problems [1,2]. *Candida albicans* is one of the most frequently recovered human pathogen from fungal infections [3,4]. *C. albicans* cell wall is a dynamic and complex structure essential to almost every aspect of pathogenicity. The cell wall inner layer act as the cell skeleton and is composed of chitin and β-glucans. The outermost layer of the *C. albicans* cell wall is composed of mannoproteins bound to the β-glucan/chitin inner layer. The recognition of *C. albicans* cell wall and subsequent host response depends on the chemical composition and linkages of the *C. albicans* cell wall polysaccharides [5,6,7,8,9]. Immune responses elicited during *C. albicans* infections are related to the host–pathogen interaction. Nevertheless, the inflammatory response elicited by fungal recognition may also be deleterious to the host by causing excessive tissue damage [10]. For instance, an exacerbated inflammatory response is often observed during neutrophil recovery in acute leukemia patients with systemic candidiasis [11]. These observations raise important questions concerning host evolution towards maintaining a balance between control of fungal burden and excessive inflammation [10,12]. A better understanding of these mechanisms may offer new insights into the pathophysiology of these infections, as well as open new possibilities for targeted anti-microbial therapy. In recent decades, β-glucans have been used in many ways to improve health conditions in various situations [13]. Administration of these polysaccharides proved to enhance efficacy of monoclonal antibodies used for cancer immunotherapy, induce faster regeneration of physically damaged tissues, reduce toxicity of bacterial endotoxin, reduce gut inflammation, enhance antibody production against mucosal antigens, and enhance inflammation defense [13,14,15,16,17,18]. Taken together, these works have demonstrated that in a context of excessive inflammatory response, administration of β-glucans can depress these deleterious responses, suggesting that β-glucans may be used as an anti-inflammatory treatment, namely, in gut inflammation and sepsis. 

The emergent application of nanotechnology is revolutionizing several aspects of modern medicine, including diagnostics and therapeutics [19,20]. Nanoparticles (NP) present a great immunomodulatory potential, as they can lead to activation or suppression of immune function [21]. Some experiments have shown that different NP trigger inflammatory responses in different ways [22,23,24]. Furthermore, the NP properties play a central role in determining the diffusion rate, the absorption of biomolecules, uptake by immune cells, biodistribution, and NP retention time. These parameters can be used to control biological functions and optimize the therapeutic applications. Here, we used bioengineered NP, functionalized with β1,3-glucans, the major component of *C. albicans* cell wall, in order to modulate the host immune recognition of *C. albicans* and reduce the inflammatory consequences of a fungal infection.

## 2. Materials and Methods

### 2.1. NP and β-glucans

Whole glucan particles (WGP) from *Saccharomyces cerevisiae*, composed mainly of β1,3-glucans ((β1→3)-Glc), were purchased from InvivoGen (San Diego, CA, USA). Carboxyl-terminated polystyrene (COOH-PS) NP (200, 80 and 26 nm) were purchased from Bangs Laboratories, Inc. (Fishers, IN, USA). All the polystyrene (PS) NP were used right after 24 h of dialysis (molecular weight cut-off (MWCO): 3500 kDa) against deionized (DI) water.

### 2.2. Chemical Characterization of Soluble β-Glucans

WGP (5 mg) were dissolved in 0.1 M NaNO_3_ solution and analyzed by Size Exclusion Chromatography (SEC) using a PL-GPC 110 chromatograph (Polymer Laboratories, Venice, CA, USA) with a pre-column PL aquagel-OH Guard 8 μm and two PL aquagel-OH MIXED 8 μm D 300 mm × 7.5 mm columns at 36 °C. A 0.1 M NaNO_3_ eluent solution was pumped at a flow rate of 0.9 mL/min. Sugar composition of soluble WGP was analyzed by gas chromatography-flame ionization detection (GC-FID) (Perkin Elmer-Clarus 400, PerkinElmer, Waltham, MA, USA) and quantified using 2-deoxyglucose as internal standard [25], as previously described [26]. The alditol acetates were evaluated in a GC-FID with a capillary column DB-225 (30 m length, 0.25 mm inner diameter and 0.15 μm film thickness). The oven temperature was: 200 °C to 220 °C at a rate of 40 °C/min (7 min), increasing to 230 °C at a rate of 20 °C/min (1 min). The temperature of the injector was 220 °C and the detector was at 230 °C. Hydrogen was used as a carrier gas at a flow rate of 1.7 mL/min [26,27].

The glycosidic-linkage composition was determined by gas chromatography-mass spectrometry (GC-MS) of partially methylated alditol acetates (PMAA) using a previously described methodology [26]. The PMAA were analyzed by GC-MS using a Shimadzu GCMS-QP2010, with a DB-1 (J&W Scientific, Folsom, CA, USA) capillary column (30 m length, 0.25 mm internal diameter, and 0.10 μm film thickness). Samples were injected in “split” mode using an injector temperature of 220 °C. The temperature program used was: 50 °C with a linear increase of 8 °C/min until 140 °C, followed by a linear increase of 0.5 °C/min until 150 °C and finally by a linear increase of 40 °C/min until 250 °C. The helium carrier gas had a flow rate of 1.84 mL/min and a column head pressure of 124.1 kPa. The GC was connected to a QP2010 Ultra (Shimadzu Corporation, Kyoto, Japan) mass quadrupole selective detector operating with an electron impact mode at 70 eV and scanning the range *m/z* 40–500, 1 s cycle, in a full scan mode acquisition.

### 2.3. Surface Functionalization of PS NP with Soluble (β1→3)-Glc 

Soluble WGP (5 mg) was dissolved in phosphate buffer (10 mM sodium phosphate, 150 mM NaCl, pH 7.5). Subsequently, sodium periodate (0.1 M) was added, and the solution was incubated for 30 min. The resultant oxidizing glucan solution was then mixed with excess of carbohydrazide (100:1) and sodium cyanoborohydride (10 mM). Dialyzed COOH-PS (1 mg/mL) was mixed with previous glucan solution and 1-ethyl-3(3-dimethylaminopropyl)carbodiimide (EDC) (1 mg) during 2 h. β-Glucan-conjugated nanoparticles (β-Glc-PS) were desalted on a PD-10 column to remove excess reactants. 

### 2.4. Size and ζ-Potential Analysis of β-Glc-PS

The hydrodynamic size of NP was determined by dynamic light scattering (DLS) on a DynaPro Plate Reader II (Wyatt Technology Corporation, Santa Barbara, CA, USA), at 37 °C, with 10 acquisitions per sample. Differential centrifugal sedimentation (DCS) was performed on DC24000 disc centrifuge (CPS Instruments, Prairieville, LA, USA), with a linear 8–24% sucrose gradient at 23,677 rotations per minute (RPM). The ζ-potential of the particles was evaluated by DLS at 25 °C with a Malvern ZetaSizer Nano ZS particle analyzer (Malvern Panalytical, Malvern, UK). Malvern Dispersion Technology Software (DTS; Malvern Panalytical, Malvern, UK) with monomodal mode data processing was used to determine average ζ-potential (mV) and error values. The analysis was conducted between 1 h and 24 h after incubation at 25 °C using 1 mg/mL, 0.1 mg/mL, and 0.01 mg/mL of NP in phosphate buffer. Each condition was set in triplicate and the measurements were analyzed with the Dynamics 7.1.7 (Wyatt Technology Corporation, Santa Barbara, CA, USA).

### 2.5. Dot Blot Analysis and Glucan Quantification of β-Glc-PS NP

For the dot blot immunoassay, 2 μL of each sample was spotted onto nitrocellulose membrane and then allowed to air dry. Each nitrocellulose membrane was incubated in blocking solution (Tris Buffer Saline-Tween (TBS-T) + 2% low fat dry milk) for 1 h at room temperature (RT). The membranes were then washed three times 10 min in TBS-T and incubated with anti-(β1→3)-Glc (1:800, Biosupplies, Yagoona, Australia) for 2 h, washed, and further incubated with polyclonal rabbit anti-mouse IgG/AP (1:500, Dako, Glostrup, Denmark) and, subsequently, with substrate solution (BCIP/NBT, Sigma-Aldrich, St. Louis, MO, USA) until spots were visible. 

The amount of (β1→3)-Glc on the surface of the NP was analyzed using a phenol-sulphuric acid assay [28].

### 2.6. FTIR Spectroscopy

For Fourier transformed infrared (FTIR) analysis, 2 mL of each NP suspension (26, 80, 200 nm β-Glc-PS NP and COOH-PS NP) was scanned using PerkinElmer Frontier FTIR (Universal Attenuated Total Reflection (ATR) Sampling Accessory; PerkinElmer, Waltham, MA, USA) with a resolution of 4 cm^−1^ in the ATR sampling mode. For each sample, 32 scans were acquired and averaged, Energy = 371 and OPD = 0.2 and recorded in the 4000–400 cm^−1^ spectral range. 

### 2.7. NP Aggregation under Physiological Conditions

PS NP were diluted with either water, PBS, RPMI plus 10% fetal bovine serum (FBS) or mouse serum (MS). The analyses were conducted between 0 h and 24 h after incubation. The hydrodynamic size of NP was measured by DLS on DynaPro Plate Reader II (Wyatt Technology Corporation, Santa Barbara, CA, USA), at 37 °C, with 10 acquisitions per sample. Before the analysis of samples, the refractive index and viscosity were adjusted according to solvent used. 

### 2.8. Mouse Serum Protein Corona Analysis

NP suspensions (0.5 mg/mL) were incubated with MS for 1 h, 12 h and 24 h. The samples were centrifuged (15 min, 14,000 rpm, 20 °C) to pellet the particle–protein complexes. The pellet was resuspended in PBS, transferred to a new vial, and centrifuged again to pellet the particle–protein complexes. This procedure was repeated three times. After the third washing step, the proteins were eluted from the particles by adding SDS-sample buffer (dH_2_O, 0.5 M Tris-HCl, Glycerol, 10% SDS, 2-mercaptoethanol, 1% bromophenol blue) to the pellet and boiling the solution. Corona proteins were separated through 10% SDS/PAGE 1D gels. Each gel run included one lane of a molecular weight ladder standard, PageRuler Prestained Protein Ladder (Thermo Scientific, Waltham, MA, USA). 

### 2.9. Yeast Cell and Culture Conditions

*C. albicans* SC5314 (ATCC MYA-2876, ATCC, Manassas, VA, USA) cells were maintained frozen (30% glycerol at −80 °C). Viable cells were obtained from cultures performed on solid yeast extract-peptone-dextrose (YPD) medium (1% yeast extract, 2% peptone, 2% glucose, 2% agar). Isolates were then grown in liquid YPD medium (37 °C, 140 rpm) in a shaking incubator to late exponential growth (14–16 h), recovered by centrifugation and washed twice in sterile phosphate-buffered saline (PBS). To prepare heat-killed (HK) cells, *C. albicans* yeast cells were grown as described before and incubated at 70 °C for 30 min in sterile PBS. 

### 2.10. Mice

BALB/c mice were bred under specific-pathogen-free conditions at the Animal Facility of the Instituto de Investigação e Inovação em Saúde (i3S), Porto, Portugal. Procedures involving mice were performed according to the European Convention for the Protection of Vertebrate Animals used for Experimental and Other Scientific Purposes (ETS 123), directive 2010/63/EU, of the European Parliament and of the council of 22 September 2010 on the protection of the animals used for scientific purposes, and Portuguese rules (DL 113/2013). Experiments were approved by the institutional board responsible for animal welfare (ORBEA) at i3S and authorization to perform the experiments was issued by the competent national authority (Direção Geral de Alimentação e Veterinária) with the reference number 014036/2019-07-24.

### 2.11. In Vitro Differentiation of BMM and BMDC

To generate bone marrow-derived macrophages (BMM) and dendritic cells (BMDC), bone marrow cells were collected from femurs and tibias of BALB/c mice by flushing with cold Roswell Park Memorial Institute (RPMI) 1640 (Sigma-Aldrich, St. Louis, MO, USA). The collected cells (1 × 106 cells/mL) were seeded onto six-well plates and incubated at 37 °C, 5% CO_2_ in RPMI supplemented with 10% fetal bovine serum (FBS) (Biowest, Nuaillé, France), penicillin (100 IU/mL)/streptomycin (100 mg/mL) (Sigma-Aldrich, St. Louis, MO, USA), l-glutamine (2 mM) (Sigma-Aldrich, St. Louis, MO, USA), 1% (*v*/*v*) 4-(2-hydroxyethyl)-1piperazineethanesulfonic acid (HEPES) buffer (Sigma-Aldrich, St. Louis, MO, USA), 5 μM 2-mercaptoethanol (Sigma-Aldrich, St. Louis, MO, USA) and 20% (*v*/*v*) J558-cell supernatant, containing murine granulocyte-macrophage colony-stimulating factor (GM-CSF), or 10% (*v*/*v*) L-cell conditioned medium (LCCM), to differentiate BMM and BMDC, respectively. The media were renewed every two days for BMDC cultures and at day 3 and 5 for BMM. On day 7, non-adherent and loosely adherent BMDC were harvested by gentle washing with PBS and BMM were carefully recovered using a cell scraper. The proportions of differentiated dendritic cells (DC) and macrophages were assessed by flow cytometry upon staining with anti-CD11c FITC-conjugated (clone HL3), anti-F4/80 PE-Cy5.5-conjugated (clone BM8), anti-MHC class II PE-conjugated (clone 2G9) and anti-CD86 PE-Cy7-conjugated (clone GL1). 

### 2.12. β-Glc-PS Cytotoxicity Analysis 

BMM and BMDC were plated in 96-well tissue culture plates (2 × 105 cells/well) and incubated at 37 °C in a humidified atmosphere in 5% CO_2_. Three different concentrations of NP were evaluated. Cell viability was assessed using the 3-[4,5-dimethylthiazol-2-yl]-2,5-diphenyltetrazolium bromide (MTT) assay. Enzymatic activity was quantified after solubilization of MTT formazan with dimethyl sulfoxide (DMSO):ethanol (1:1) solution. Absorbance was then measured at 570 nm. Untreated cells were used as viability control (100%). Normal, apoptotic, and necrotic cells were determined using an Annexin V-FITC/propidium iodide (PI) assay kit (eBioscience, San Diego, CA, USA) according to manufacturer’s instructions. The flow cytometry analysis was performed within 15 min in a BD FACSCanto^TM^II (BD Biosciences, Franklin Lakes, NJ, USA), using the FlowJo software (version 10.0.7; FlowJo, Ashland, OR, USA).

### 2.13. Phagocytosis Quantification Assay

The phagocytosis of *C. albicans* by BMDC and BMM was analyzed by flow cytometry using a previously described methodology [29]. BMDC and BMM suspensions were incubated with Sytox Green-labeled *C. albicans* HK at a MOI (Multiplicity of infection) of 1:5 (1 BMDC/BMM to 5 yeasts) and treated with 10 μg/mL NP for 30 min, at 37 °C and 5% CO_2_. Cells were analyzed by flow cytometry in a BD FACSCanto^TM^ II, using the FlowJo software (version 10.0.7; FlowJo, Ashland, OR, USA). The percentage of yeasts interacting with immune cells was calculated from dot plot analysis of Sytox Green vs. PI fluorescence intensities.

### 2.14. Evaluation of ROS Production

Reactive oxygen species (ROS) production was evaluated using the Superoxide Detection Kit for flow cytometry (Enzo Life Sciences, Farmingdale, NY, USA). *C. albicans* HK yeast cells were incubated with BMM or BMDC (MOI of 1 BMDC/BMM: 5 yeasts) and treated at same time with 10 μg/mL NP or 10 μg/mL WGP. As a positive control, BMM and BMDC were treated with 100 nM PMA (Sigma-Aldrich, St. Louis, MO, USA). Plates were incubated for 15 min at 37 °C and 5% CO_2_. Upon incubation, cells were prepared according to manufacturer’s instructions. Samples were immediately analyzed in a BD FACSCanto II and acquired data were analyzed by using FlowJo software (version 10.0.7; FlowJo, Ashland, OR, USA). 

### 2.15. Cytokine Quantification by Sandwich ELISA 

BMDC were incubated for 24 h with yeast cells at a MOI of 1 BMDC: 5 Yeasts. Cultures were treated with 10 μg/mL of NP or 10 μg/mL of WGP.

Cytokine levels in cell culture supernatants and serum samples were evaluated by sandwich enzyme-linked immunosorbent assay (ELISA) using commercial kits, according to the manufacturer’s instructions (Mouse IL-6, IL-12p70 ELISA Ready-SET-Go!^®^, eBioscience, San Diego, CA, USA; Mouse TNF-α and IL-1β MAX Standard, Biolegend, San Diego, CA, USA).

### 2.16. Statistical Analysis

All experiments were performed at least in triplicate (n ≥ 3). Data are reported as means ± SD and were analyzed by one-way ANOVA and Tukey’s post hoc test using GraphPad Prism (Version 7.0; GraphPad Software, San Diego, CA, USA). Statistically significant results were defined as follows: * *p* < 0.05, ** *p* < 0.01, *** *p* < 0.001 and **** *p* < 0.0001.

## 3. Results and Discussion

### 3.1. Characterization of Soluble Whole Glucan Particle (WGP)

Soluble WGP was used to functionalize carboxyl-terminated polystyrene nanoparticles (COOH-PS NP) sized 26, 80 and 200 nm. The WGP was characterized according to their molecular weight (MW) distribution, neutral sugars, and glycosidic-linkage analyses (Figure S1, Table 1 and Table 2).

Neutral sugar analysis (Table 1) showed that soluble WGP was composed of 93.5% glucose. As expected, since soluble WGP is obtained from *S. cerevisiae* cell wall extractions, minor contaminations with other cell wall sugars, like mannose and arabinose, were also detected. The used soluble WGP was composed mostly of (1,3)-linked glucose (53.5%) followed by 12.6% of (1,6)-linked glucose (Table 2) distributed into two populations with a MW of 50 and 280 kDa (Figure S1), where the highest MW corresponds mainly to (β1→3)-Glc and the lowest MW corresponds mainly to (β1→3)-Glc and (β1→6)-Glc (Table S1 and Figure S2).

### 3.2. Production and Characterization of β-Glc-PS

**Figure 1:**
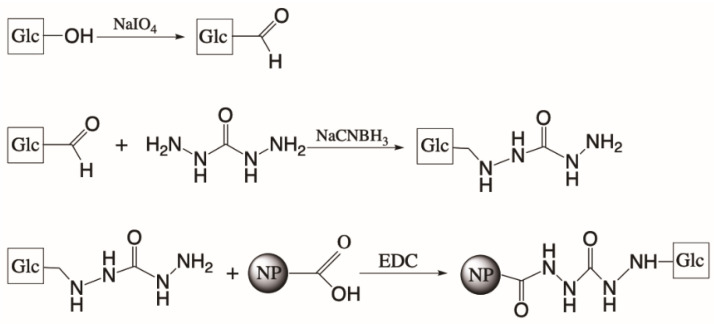
Schematic representation of the chemical conjugation of soluble WGP onto COOH-PS NP surface.

Directional conjugation of WGP onto COOH-PS was achieved through carbonyl-reactive chemistry (Figure 1). Three different NP sizes (200, 80 and 26 nm) were chosen as the size of NP may influence WGP delivery to and recognition by cells of the immune system. Protein binding is dependent on NP size as shown for unconjugated COOH-PS NP in mouse and human serum [30,31], which together with the size itself can affect biodistribution and secretion pathways [32,33].

Soluble WGP were oxidized with periodate in order to create aldehyde residues between C2−C3 and C3-C4 bonds of the non-reducing end or (β1→6)-Glc residues [34]. This means that the polymer of lower molecular weight was more prone to be modified than the one with the highest molecular weight which, due to its richness in (β1→3)-Glc residues, resistant to periodate oxidation, and lower content in terminal residues. These oxidized β-glucans were coupled to carbohydrazide and this bond was further stabilized by reduction with sodium cyanoborohydride. β-glucan-hydrazide molecules were incubated with COOH-PS NP in the presence of EDC for a complete conjugation [35].

The size of conjugated NP was determined by DLS and DCS (Table 3 and Figure S3). All NP showed increased radius after β-glucan conjugation. Moreover, all β-Glc-PS NP presented less negative surface charge than naked COOH-PS NP counterparts (Table 3). This charge shift was strongly related with the presence of (β1→3)-Glc onto PS NP surface and the consequent reduction of surface carboxyl groups. 

**Figure 2:**
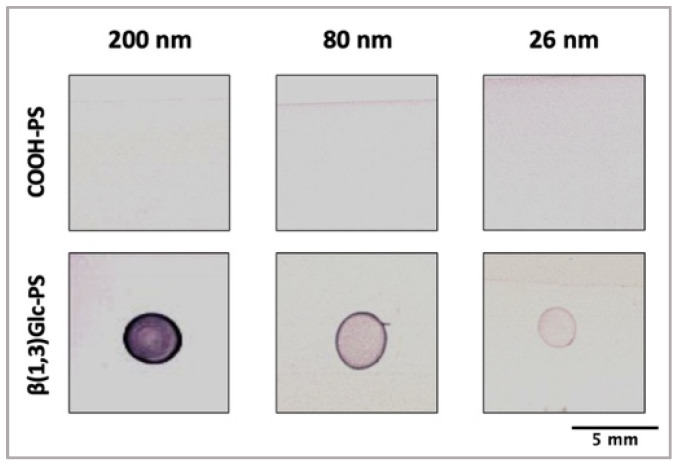
Analysis of (β1,3)-glucan onto NP surface after conjugation. Dot Blot analysis of COOH-PS and β-Glc-PS. After conjugation protocol, 0.5 mg/mL of β-Glc-PS and COOH-PS NP were spotted (5 μL) on nitrocellulose membranes and incubated with (β1,3)-glucan-specific mAb.

The amount of β-glucans on the surface of NP was determined using the phenol-sulphuric acid assay (Table 4) [28]. The average β-glucan content increased as the NP size increased. These quantifications were in accordance with dot blot analysis using (β1→3)-Glc-specific mAb (Figure 2). Although 200 nm NP presented lower surface area per mass and less possible conjugation sites available (1.0 × 10^20^ COOH groups/mL) when compared with 80 nm (1.3 × 10^20^ COOH groups/mL) and 26 nm (3.4 × 10^20^ COOH groups/mL), the 200 nm NP bound more (β1→3)-Glc than the other NP used. Size-related (β1→3)-Glc conjugation efficacy could be associated with NP size and curvature effect. As previously reported, lower NP curvature facilitates ligand–ligand interactions and hence a higher packing density [36,37].

**Figure 3:**
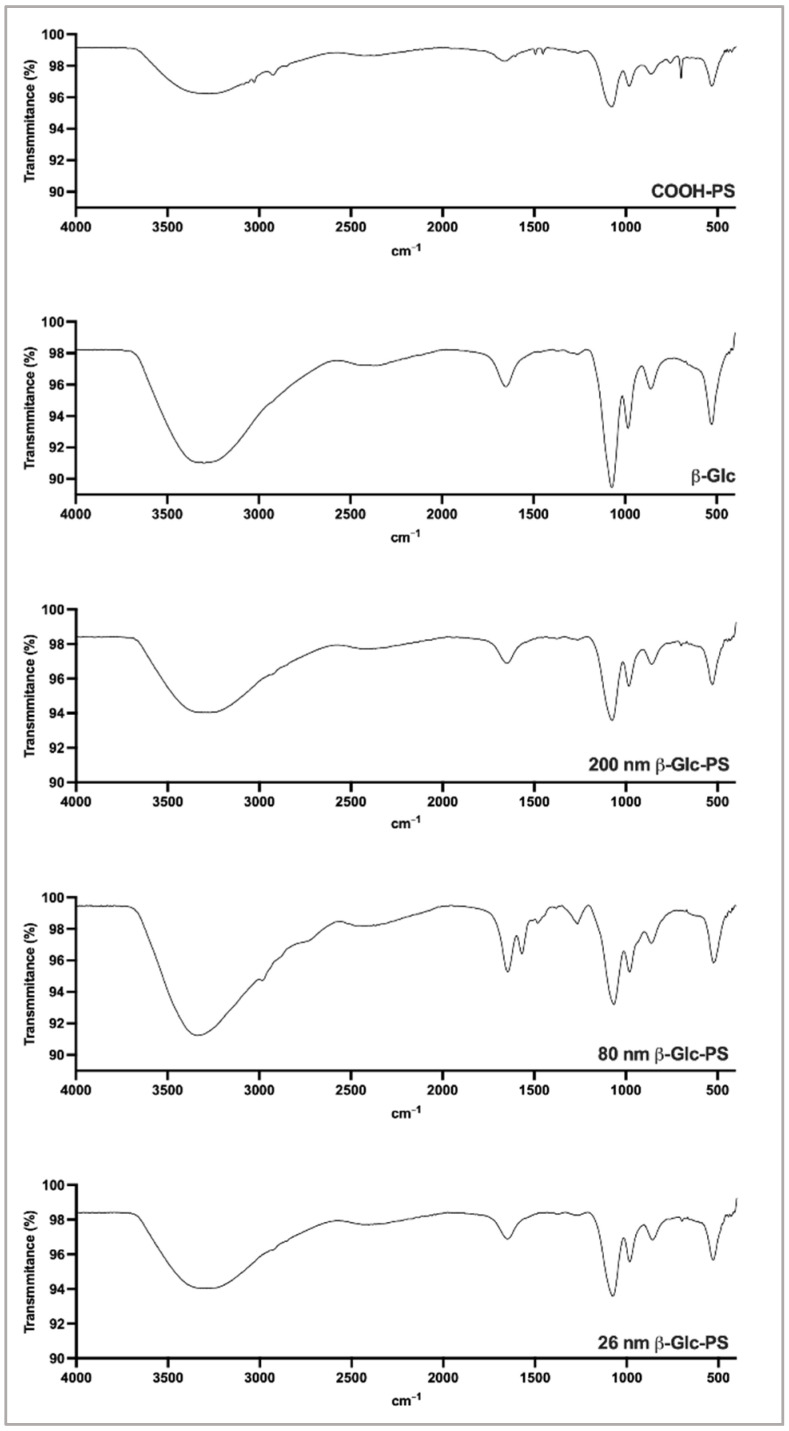
Fourier transformed infrared (FTIR) spectra of COOH-PS, soluble β-Glc and β-Glc-PS NP. Sample suspensions were scanned using PerkinElmer Frontier FTIR (Universal ATR Sampling Assessory) with a resolution of 4 cm^−1^ in ATR sampling mode. Each condition was set in triplicate.

The successful covalent conjugation of (β1→3)-Glc onto PS NP surface was also confirmed using Fourier transform infrared (FTIR) spectroscopy (Figure 3). The β-Glc-PS spectra showed a band at ~1650 cm^−1^ (amine I) and 1550 cm^−1^ (amine II), indicating the successful conjugation of β-glucans onto PS surface [38,39,40,41]. Moreover, the band at the fingerprint region of 950–1200 cm^−1^, due to the stretching vibration associated with the C-O-C and C-O-H groups of polysaccharides [27], also confirming the existence of β-glucans on the NP surface [42].

The formation of protein corona modifies the physical and chemical NP properties, namely their hydrodynamics size and surface charge. Moreover, the opsonization of NP by protein corona formation has a central role in the recognition of NP by immune cells. To understand the corona profile of NP in a physiological environment, NP were incubated with MS and the protein corona formation was analyzed by SDS-page (Figure S4). The protein binding after incubation with MS was compared between the three sizes of NP. In previous work, we demonstrated a size-dependent protein corona formation around COOH-PS NP after MS incubation. However, after the NP surface conjugation with soluble β-glucans, no significant differences in protein corona were observed between the three β-Glc-PS NP sizes. 

Aggregation of NP was also evaluated after incubation with MS and RPMI + 10% FBS (Figure S5). The 26 nm β-Glc-PS NP showed higher size variations after incubation with RPMI + 10% FBS and MS when compared with 80 nm and 200 nm NP. The aggregation is not obviously associated with differences in NP protein corona. However, the structure of absorbed proteins could be different among NP sizes affecting aggregation and consequently affecting biodistribution, immune recognition, and NP uptake [43].

### 3.3. Biocompatibility of β-Glc-PS with BMDC and BMM 

**Figure 4:**
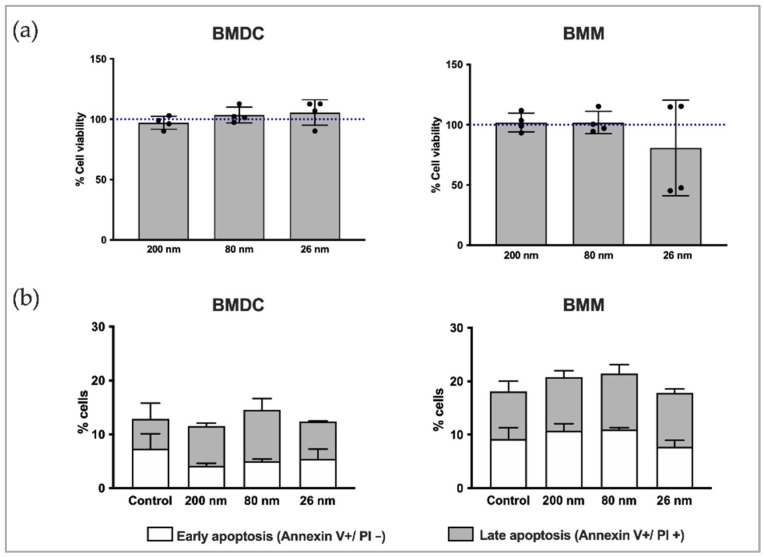
Cell viability of BMDC and BMM treated with β-Glc-PS. BMDC (left) and BMM (right) were stimulated with three different-sized β-Glc-PS for 24 h at a concentration of 10 μg/mL and analyzed in a (**a**) MTT assay and by (**b**) FITC-annexin V staining, using flow cytometry. Samples were acquired on FACSCantoII (BD Biosciences) and data analyzed with FlowJo software (Version 10.0.7). Each black dot corresponds to the mean value of three technical replicates of an independent experiment.

Biocompatibility of β-Glc-PS was assessed with MTT assay and Annexin V/Propidium Iodide (PI) staining using BMDC and BMM (Figure 4a,b). A slight decrease in cell viability was detected when using 26 nm NP while no significant effects were detected using 80 and 200 nm NP. This decrease in cell viability could be related with the high aggregation observed for 26 nm NP when incubated in serum-containing media which might influence its cell interaction and induce cytotoxicity (Figure S5). For the same mass of NP, smaller NP present a higher surface area and thus more available surface to interact with macromolecules, in addition to higher aggregation as already mentioned. Particle size and available surface area may thus contribute to the higher cell toxicity of the 26 nm NP [44]. The protein corona composition does not seem likely to have had a role in toxicity since after conjugation with β-glucans, as all the different sized NP showed similar protein corona profile (Figure S4). Although we cannot exclude that aggregation could influence the NP physical properties and decrease the surface area available, the complexity of 26 nm NP aggregates and the high polydispersity and heterogenicity can still result in cell toxicity since they cannot be assumed to behave like single and stable larger-size NP.

### 3.4. Effect of β-Glc-PS on Phagocyte Function

Phagocytosis plays a critical role in innate immunity, promoting the removal and killing of pathogens, and triggering the adaptive immune response [45]. 

**Figure 5:**
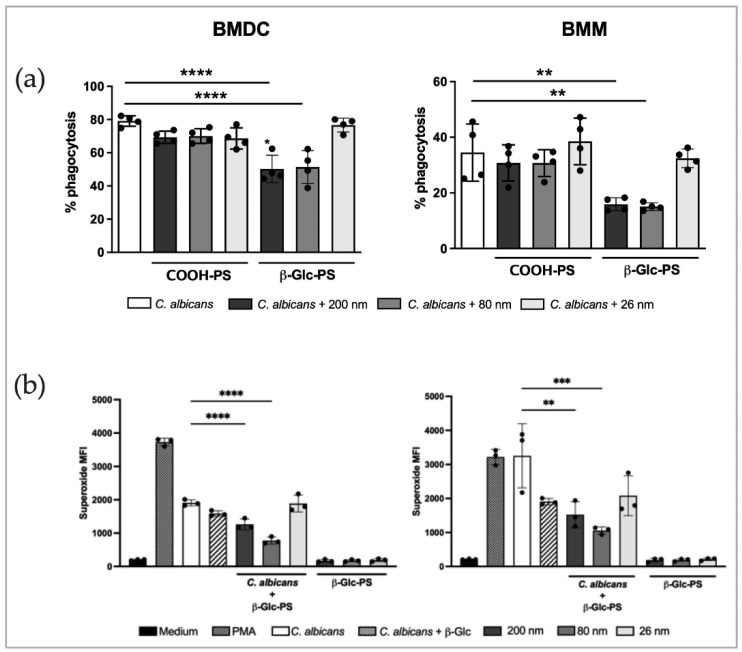
Analysis of β-Glc-PS treatment on phagocyte function. (**a**) Quantification of yeast phagocytosis by BMDC and BMM was assessed by flow cytometry. Immune cells were incubated for 30 min with heat-killed *C. albicans* SC5314 (MOI 1 BMDC/BMM: 5 yeasts) labeled with Sytox Green plus 80 nm β-Glc-PS (10 μg/mL) and stained with Propidium Iodide prior to analysis. (**b**) Production of ROS was assessed by using Superoxide Detection kit. BMM and BMDC incubated for 15 min with heat-killed *C. albicans* SC5314 at a MOI 1:5, in the presence of 200, 80 or 26 nm β-Glc-PS NP (10 μg/mL) and analyzed by flow cytometry. Samples were acquired on FACSCantoII (BD Biosciences) and data analyzed with FlowJo software (Version 10.0.7). Bars correspond to means ± SD. Each condition was set in triplicate. Each black dot corresponds to the mean value of the three technical replicates of an independent experiment. One-way ANOVA with Tukey’s post Hoc test, (** *p* < 0.01; *** *p* < 0.001; **** *p* < 0.0001). The complete statistical analysis data is presented in the Supplementary Material (Table S2).

Phagocytosis assays were performed in vitro using BMDC and BMDC incubated with *C. albicans* and treated with different sized β-Glc-PS. Naked NP (COOH-PS) were used as controls to assess a possible effect of the used polymer. Treatment with 200 or 80 nm β-Glc-PS induced a reduction of yeast phagocytosis, observed in both BMDC and BMM (Figure 5a, gating strategy shown in Figure S6). No reduction in yeast phagocytosis was observed using 26 nm NP, which could indicate a size-dependent effect. Also, no effect was observed on phagocytic function using COOH-PS.

Production of reactive oxygen metabolites is considered a major antifungal mechanism in phagocytes [46,47]. Therefore, we studied the ability of β-Glc-PS to modulate ROS production (Figure 5, Figure S7 and Table S2). As shown in Figure 5b, lower levels of ROS were produced in *C. albicans*-challenged BMDC and BMM treated with β-Glc-PS when compared with non-treated cells. This could be related to the observed reduction of β-glucan-mediated *C. albicans* phagocytosis (Figure 5a). These results indicate that β-Glc-PS may interfere with effector mechanisms used by phagocytic cells in the course of *C. albicans* infections. The immobilization of β-Glc onto NP surface showed to be more effective in reducing ROS production than did free soluble β-Glc treatment. No significant ROS production was detected in non-infected cells treated with β-Glc-PS alone, which reinforces the absence of a nanoparticle cytotoxic effect [48]. COOH-PS had no effect on ROS production by BMM and BMDC after *C. albicans* stimulation (Figure S7).

### 3.5. β-Glc-PS NP Reduced the Proinflammatory Response of Dendritic Cells Induced by C. albicans

**Figure 6:**
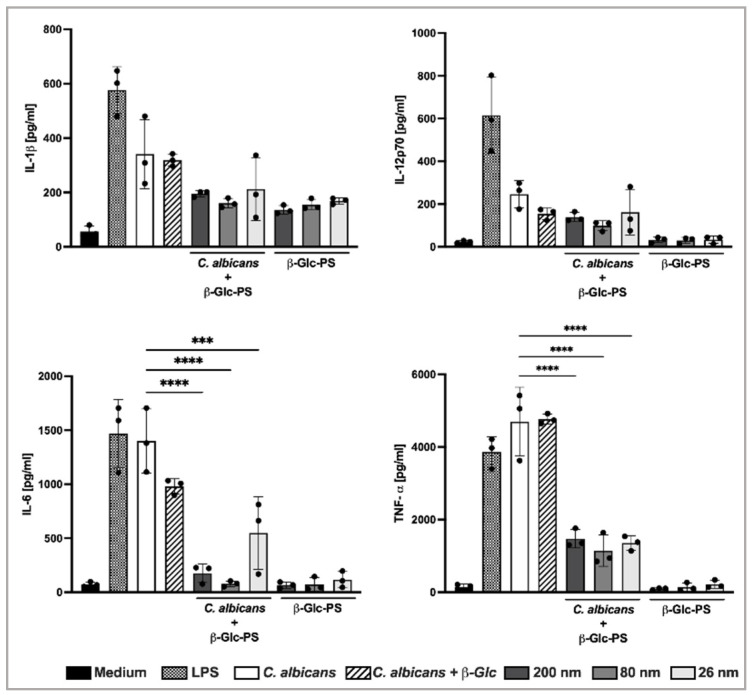
Quantification of cytokines in supernatants of BMDC cultures. The levels of TNF-α, IL-1β, IL-6 and IL-12p70 secreted by BALB/c mice BMDC were quantified by sandwich ELISA. Immune cells were incubated with live *C. albicans* SC5314 at a MOI of 1:5 (1 DC: 5 yeast cells) for 24 h. The 80 nm β-Glc-PS (10 μg/mL) were added 1 h prior to infection. Each condition was set in triplicate. Each black dot corresponds to the mean value of the three technical replicates of an independent experiment. Bars correspond to means ± one SD. One-way ANOVA with Tukey’s post hoc test. (*** *p* < 0.001; **** *p* < 0.0001). The complete statistical analysis data is presented in the Supplementary Material (Table S3).

Following recognition of pathogens by Pathogen Recognition Receptors, distinct signaling pathways are induced. These pathways act through a cross-regulation mechanism, which results in the production of proinflammatory cytokines [49]. The induction of proinflammatory cytokines is an important component of antifungal host defense [50]. Thus, the levels of TNF-α, IL-1β, IL-6, and IL-12p70 were quantified in the culture supernatants of *C. albicans*-challenged BMDC treated and untreated with β-Glc-PS. All the cultures treated with β-Glc-PS showed a marked reduction of TNF-α, IL-1β, IL-6, IL-12p70 levels (Figure 6, Figure S8 and Table S3). β-Glc-PS anti-inflammatory effect showed size-dependency. The 26 nm β-Glc-PS NP was the least effective size. No significant reduction in cytokines levels was observed in BMM and BMDC cultures challenged with *C. albicans* and treated with COOH-PS (Figure S8).

The β-Glc-PS NP treatment showed size dependency. In fact, 80 nm β-Glc-PS was the most biologically effective size. Smaller NP presents a higher surface area per mass, and thus more available surface to interact with cells and cellular components, in addition to higher aggregation and less stability. This may therefore have contributed to the less effective results obtained with 26 nm β-Glc-PS NP (Figure S4) [44]. The curvature could potentially affect the density of conjugated WGP as well as the angles between the WGP chains affecting the binding to Dectin-1 receptor [35,36]. Moreover, the diffusion rate, which increases with decreasing particle size, is also an important factor for ligand receptor recognition [51]. The immobilization of (β1→3)-Glc onto NP surface enhances the anti-inflammatory effect of these polysaccharides when compared with treatment with free soluble (β1→3)-Glc.

## 4. Conclusions

*C. albicans* asymptomatically colonizes more than 30% of individuals in a population at any given time. However, when the host immune system of the carrier individuals is weakened, this fungus can cause both mucosal and systemic infections. Some risk factors, such as neutropenia, central venous catheters, or systemic antibiotic exposure, may predispose individuals to invasive and even life-threatening systemic candidiasis. Invasive *C. albicans* infections can result in an uncontrolled hyper-inflammatory response, leading to severe host damage. With this study we showed that β-glucan-functionalized nanoparticles could down-modulate the proinflammatory response of host immune cells induced by *C. albicans,* in a size-dependent manner. This NP-based approach showed a promising therapeutic potential, extending the described potential of (β1→3)-Glc in host inflammation control.

## Figures and Tables

**Table 1:** Characterization of soluble WGP. Neutral sugar analysis of soluble WGP. Carbohydrate composition of (β1,3)-Glucan (WGP) was determined by GC-FID and quantified using 2-deoxyglucose as internal standard. Mean results are presented in molecular percentage (mol %) and mass concentration (mg/g). Each condition was set in triplicate.

	WGP	
	mol %	C (mg/g)
Glucose	93.35	607.91
Mannose	0.58	3.54
Arabinose	0.07	0.33

**Table 2:** Characterization of soluble WGP. Glycosidic linkage composition (molecular percentage) of soluble WGP. The glycosidic-linkage composition of soluble WGP was determined by GC-MS of partially methylated alditol acetates. Man—Mannose; Glc—Glucose; GlcNAc—*N*—Acetylglucosamine Each condition was set in triplicate.

	WGP (mol %)
t-Man	0.80
2,3-Man	0.21
**Total Man**	**1.01**
t-Glc	20.60
3-Glc	53.53
4-Glc	1.88
6-Glc	12.57
3,4-Glc	1.00
2,3-Glc	3.70
3,6-Glc	3.81
2,3,4-Glc	1.28
2,3,6-Glc	0.18
**Total Glc**	**98.55**
4-GlcNAc	1.4

**Table 3:** Physical properties of polystyrene NP before (COOH-PS) and after conjugation with (β1,3)-glucans (β-Glc-PS). The average hydrodynamic size of COOH-PS and β-Glc-PS NP was evaluated through dynamic light scattering (DLS) at a concentration of 0.5 mg mL^−1^. PDI values were determined by DLS using DynaPro Plate Reader II. Zeta-potential analysis of COOH-PS and β-Glc-PS was performed using Malvern Zetasizer Nano ZS particle analyser. Surface area valeues were obtained from manufacturer. Values represent mean ± SD from three independent experiments.

Manufacturer Nominal Size (nm)	Surface Conjugation	Diameter (nm)	PDI	ζ-Potential (mV)	Surface Area (μm^2^ g^−1^)
200	COOH-PS	189.9 ± 3.3	0.02 ± 0.01	−35.2 ± 2.0	2.9 × 10^13^
	β-Glc-PS	199.3 ± 3.6	0.03 ± 0.02	−12.2 ± 0.4	
80	COOH-PS	87.1 ± 0.7	0.09 ± 0.02	−35.8 ± 1.4	7.1 × 10^13^
	β-Glc-PS	92.0 ± 0.9	0.08 ± 0.01	−12.9 ± 1.8	
26	COOH-PS	26.1 ± 1.3	0.36 ± 0.01	−27.3 ± 1.3	2.2 × 10^14^
	β-Glc-PS	47.5 ± 0.1	0.57 ± 0.02	−8.6 ± 0.6	

**Table 4:** β-glucan content on 0.5 mg/mL NP surface after conjugation was quantified according to Dubois et al. Values represent means ± SD.

β-Glc-PS NP	Glucan Concentration (μg/mL)
200 nm	4.44 ± 1.67
80 nm	2.97 ± 1.10
26 nm	1.41 ± 0.32

## Data Availability

The datasets generated and/or analyzed during the current study are available from the corresponding author on reasonable request.

## Supplementary Materials

The following supporting information can be downloaded at: https://www.mdpi.com/article/10.3390/nano12142475/s1, Figure S1: Characterization of soluble WGP. Chromatogram obtained by gel permeation chromatography (GPC); Figure S2. Light Scattering analysis of WGP SEC separation. Table S1. Glycosidic linkage composition of WGP fractions after SEC separation. Figure S3: Physical properties of NP after conjugation with β-(1,3)-glucans; Figure S4: Protein corona of β-Glc-PS after mouse serum incubation; Figure S5: Hydrodynamics radius of 200 nm, 80 nm and 26 nm in biological conditions; Figure S6: Representative FACS gating strategy of phagocytosis assay; Figure S7: Effect of COOH-PS NP treatment in BMDC and BMM ROS production; Table S2: Statistical analysis of BMDC and BMM ROS production; Figure S8: Effect of COOH-PS NP treatment in BMDC pro-inflammatory cytokine production. Table S3: Statistical analysis of BMDC proinflammatory cytokine production.
