# Supplementary material for: β-Glucan-Functionalized Nanoparticles Down-Modulate the Proinflammatory Response of Mononuclear Phagocytes Challenged with Candida albicans
Source: Nanomaterials (Basel). 2022 Jul 19;12(14):2475. doi: 10.3390/nano12142475 (PMC9317568; doi:10.3390/nano12142475)
Supplement: Supplementary file 1 [file nanomaterials-12-02475-s001.zip › nanomaterials-1797579-supplementary.pdf]

nanomaterials

Supplementary Materials for:

β-Glucan-Functionalized Nanoparticles Down-Modulate the Proinflammatory Response of Mononuclear Phagocytes Challenged with *Candida albicans*

Tânia Lima [1,2,3,*], Stefán B. Gunnarsson [4,5], Elisabete Coelho [6], Dmitry V. Evtuguin [7], Alexandra Correia [1,2,3], Manuel A. Coimbra [6], Tommy Cedervall [4,5] and Manuel Vilanova [1,2,3]

1 I3S-Instituto de Investigação e Inovação em Saúde, Universidade do Porto, 4200-135 Porto, Portugal; alexandra.correia@ibmc.up.pt (A.C.); vilanova@icbas.up.pt (M.V.)
2 IBMC-Instituto de Biologia Molecular e Celular, Universidade do Porto, 4200-135 Porto, Portugal
3 ICBAS-Instituto de Ciências Biomédicas de Abel Salazar, Universidade do Porto, 4050-313 Porto, Portugal
4 Department of Biochemistry and Structural Biology, Lund University, 221 00 Lund, Sweden; sbragig@gmail.com (S.B.G.); tommy.cedervall@biochemistry.lu.se (T.C.)
5 NanoLund, Center for Nanoscience, Lund University, 221 00 Lund, Sweden
6 LAQV-REQUIMTE, Departamento de Química, Universidade de Aveiro, 3810-193 Aveiro, Portugal; ecoelho@ua.pt (E.C.); mac@ua.pt (M.A.C.)
7 CICECO, Department of Chemistry, University of Aveiro, 3810-193 Aveiro, Portugal; dmitrye@ua.pt
* Correspondence: tania.lima@i3s.up.pt

Citation: Lima, T.; Gunnarsson, B.S.; Coelho, E.; Evtuguin, D.V.; Correia, A.; Coimbra, M.A.; Cedervall, T.; Vilanova, M. β-Glucan-Functionalized Nanoparticles Down-Modulate the Proinflammatory Response of Mononuclear Phagocytes Challenged with *Candida albicans*. *Nanomaterials* **2022**, *12*, 2475. https://doi.org/10.3390/nano12142475

Academic Editor: Goran Kaluđerović, Nebojša Pantelić

Received: 16 June 2022
Accepted: 10 July 2022
Published: 19 July 2022

Publisher's Note: MDPI stays neutral with regard to jurisdictional claims in published maps and institutional affiliations.

Copyright: © 2022 by the authors. Submitted for possible open access publication under the terms and conditions of the Creative Commons Attribution (CC BY) license (https://creativecommons.org/licenses/by/4.0/).

1. Material and Methods

1.1. Size exclusion chromatography (SEC)

Preparative size exclusion chromatography was performed on a HiPrep Sephacryl S-300 or Bio-Gel P2 using a peristaltic pump with a flow rate of 0.50 mL/min of degasified distilled water at room temperature. The use of distilled water as eluent allows direct analysis of the fractions by light scattering. Exclusion and total volume were calibrated with 1 mL of solution containing 2 mg of blue dextran and 1 mg of glucose, respectively. Standard dextrans of 12 kDa, 50 kDa, 150 kDa and 270 kDa were also used for column calibration. The samples were dissolved in 1 mL of distilled water and loaded on the column previously equilibrated with distilled water. Fractions (1.5 mL) were continuously collected and measured by light scattering (Sedex55, S.E.D.E.R.E France), using 90 μL of sample. Measurements were carried with a peristaltic pump to the evaporative light scattering and detection was performed with a flow rate of 1 mL/min at 57°C, N^2 pressure of 2.0 bar.

2. Results

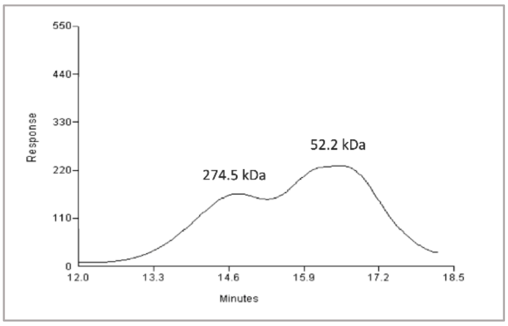

Figure S1. Characterization of soluble WGP. Chromatogram obtained by gel permeation chromatography (GPC) WGP analyzed using a S350 PL aquagel-OH column. Eluent: 0.2M NaNO3 0.01M NaH2PO4 pH 7. Eluent rate: 1.0 ml/min. Calibration curve Log (MP) =10.66-0.366 Tr.

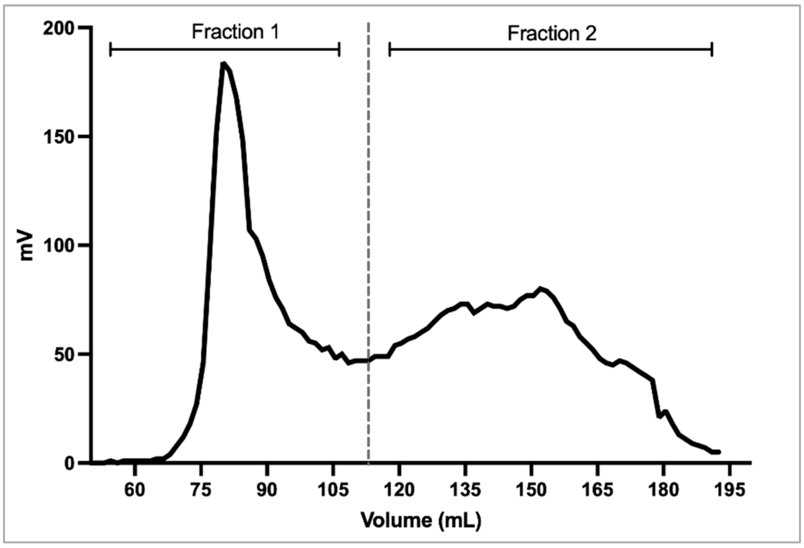

Figure S2. Light Scattering analysis of WGP SEC separation. Preparative separation performed on a HiPrep Sephacryl S-300 using Blue Dextran (exclusion volume) and glucose (inclusion volume) for column calibration and dextran with 12, 50, 150 and 270 kDa as standards. Samples measurements were performed using light scattering system analysis.

Table S1. Glycosidic linkage composition (molecular percentage) of WGP fractions after SEC separation. The glycosidic-linkage composition was determined by GC-MS of partially methylated alditol acetates. Man- Mannan; Glc- Glucose.

	**Fraction 1 (274.5 kDa)**	**Fraction 2 (52.2 kDa)**
t-Man	0.44	1.08
Total	0.44	1.08
t-Glc	17.98	22.32
**3-Glc**	**67.27**	**52.83**
4-Glc	2.05	1.62

**6-Glc**	**5.81**	**16.38**
3,4-Glc	0.4	0
2,3-Glc	1.07	0.45
3,6-Glc	4.17	4.88
4,6-Glc	0.25	0.27
2,3,6-Glc	0.07	0.06
3,4,6-Glc	0.45	0.11
Total	93.96	96.26

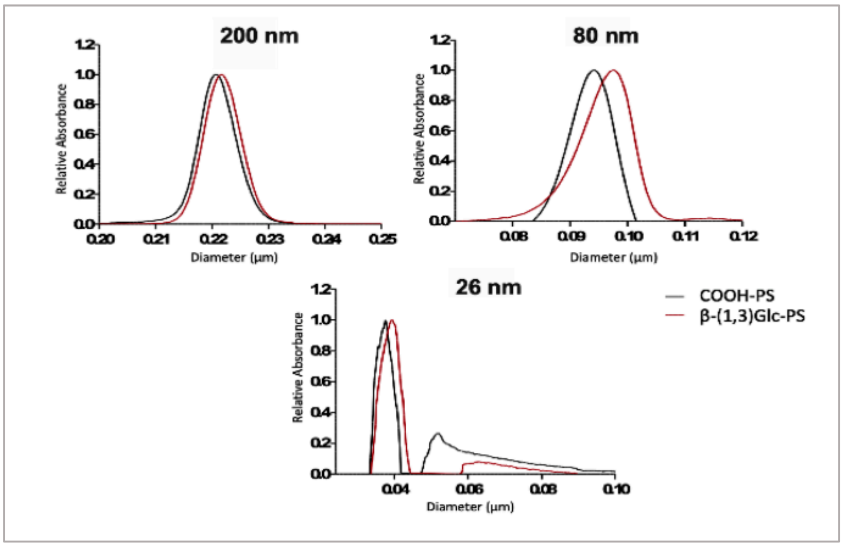

Figure S3. Physical properties of NP after conjugation with (β1→3)-Glc. The hydrodynamic size of β-Glc-PS was evaluated before (COOH-PS) and after (β-Glc-PS) functionalization through DCS using sucrose gradient. Each condition was set in triplicate.

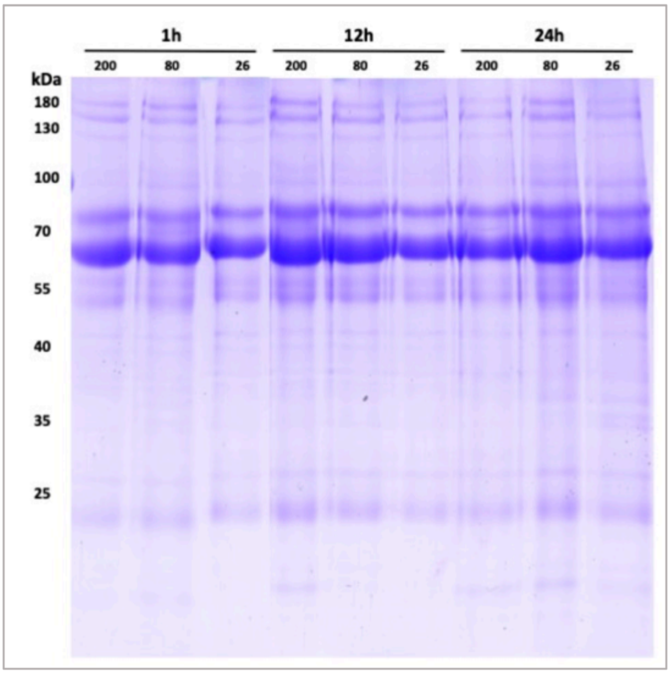

Figure S4. Protein corona of β-Glc-PS after mouse serum incubation. Nanoparticles (200, 80 and 26 nm) were incubated at 0.5 mg/ml (mass normalized samples) with MS during 1h, 12h and 24h at 37ºC. Nanoparticles were separated from free proteins by centrifugation and proteins adsorbed to β-Glc-PS nanoparticles were resolved by 10% SDS-page gel and stained with Coomassie.

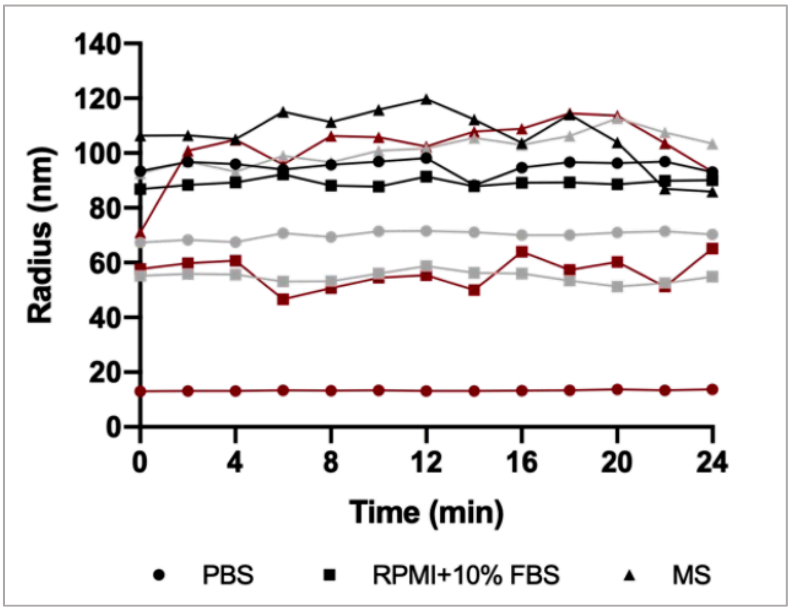

Figure S5. Hydrodynamics radius of 200 nm, 80 nm and 26 nm in biological conditions. β-Glc-PS in PBS (control), RPMI supplemented with 10% of FBS and MS. Measurements by DLS were conducted automatically every 120 min, during 24h at 37°C. The analysis was made adjusting the refractive index and viscosity to which solvent (Red- 26 nm; Grey- 80 nm; Black- 200 nm).

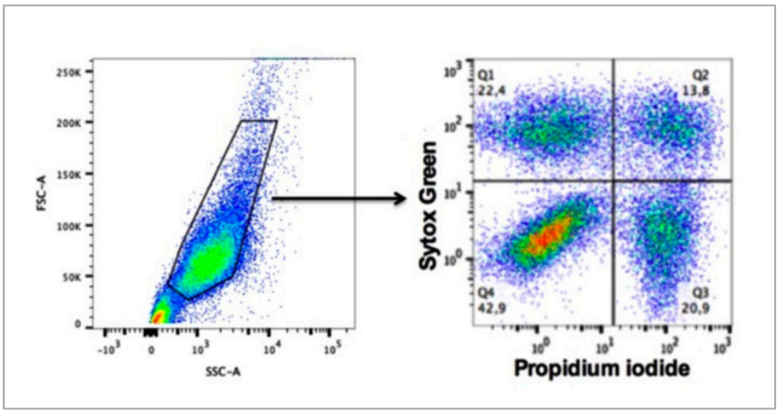

Figure S6. Quantification of yeast phagocytosis by BMDC and BMM was assessed by flow cytometry. Representative FACS gating strategy of phagocytosis assay. After gating the BMDC or BMM (left) the quantification of four sub-populations was performed (right). Q1: BMDC/BMM only with internalized yeasts, Q2: BMDC/BMM with internalized and adhered yeasts, Q3: BMDC/BMM only with adhered yeasts or dead BMDC/BMM, Q4: Live BMDC/BMM without interaction with yeasts.

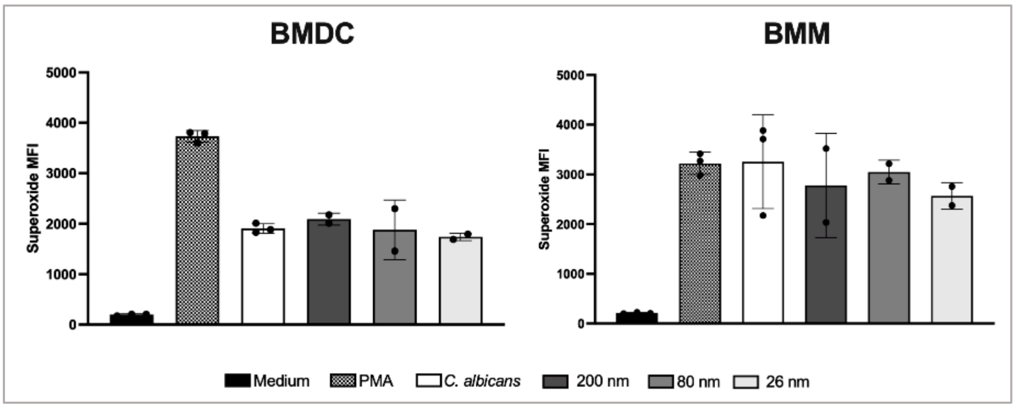

Figure S7. Effect of COOH-PS NP treatment in BMDC and BMM ROS production. BMM and BMDC were incubated for 15 min with *C. albicans* SC5314 at a multiplicity of infection (MOI) 1:5, in the presence of 200 nm, 80 nm and 26 nm COOH-PS NP (10 μg/mL) and analyzed by flow cytometry. Bars correspond to means ± SD. Data were analyzed using One-way ANOVA with Tukey's post Hoc test.

Table S2. Statistical analysis of BMDC and BMM ROS production. One-way ANOVA with Tukey's post Hoc test (**P*<0.05; ***P*<0.01; ****P*<0,001; *****P*<0.0001; ns- not significant.

Tukey's multiple comparisons test	BMDC	BMM
Medium vs. PMA	****	****
Medium vs. *C. albicans*	****	****
Medium vs. *C. albicans* + β-Glc	****	***
Medium vs. *C. albicans* + 200 nm β-Glc-PS	****	*
Medium vs. *C. albicans* + 80 nm β-Glc-PS	***	ns
Medium vs. *C. albicans* + 26 nm β-Glc-PS	****	***
Medium vs. 200 nm β-Glc-PS	ns	ns

Medium vs. 80 nm β-Glc-PS	ns	ns
Medium vs. 26 nm β-Glc-PS	ns	ns
PMA vs. *C. albicans*	****	ns
PMA vs. *C. albicans* + β-Glc	****	*
PMA vs. *C. albicans* + 200 nm β-Glc-PS	****	***
PMA vs. *C. albicans* + 80 nm β-Glc-PS	****	****
PMA vs. *C. albicans* + 26 nm β-Glc-PS	****	*
PMA vs. 200 nm β-Glc-PS	****	****
PMA vs. 80 nm β-Glc-PS	****	****
PMA vs. 26 nm β-Glc-PS	****	****
C. albicans vs. *C. albicans* + β-Glc	ns	**
C. albicans vs. *C. albicans* + 200 nm β-Glc-PS	****	***
C. albicans vs. *C. albicans* + 80 nm β-Glc-PS	****	****
C. albicans vs. *C. albicans* + 26 nm β-Glc-PS	ns	*
C. albicans vs. 200 nm β-Glc-PS	****	****
C. albicans vs. 80 nm β-Glc-PS	****	****
C. albicans vs. 26 nm β-Glc-PS	****	****
C. albicans + β-Glc vs. C. albicans + 200 nm β-Glc-PS	ns	ns
C. albicans + β-Glc vs. C. albicans + 80 nm β-Glc-PS	****	ns
C. albicans + β-Glc vs. C. albicans + 26 nm β-Glc-PS	ns	ns
C. albicans + β-Glc vs. 200 nm β-Glc-PS	****	***
C. albicans + β-Glc vs. 80 nm β-Glc-PS	****	***
C. albicans + β-Glc vs. 26 nm β-Glc-PS	****	***
C. albicans + 200 nm β-Glc-PS vs. C. albicans + 80 nm β-Glc-PS	**	ns
C. albicans + 200 nm β-Glc-PS vs. C. albicans + 26 nm β-Glc-PS	****	ns
C. albicans + 200 nm β-Glc-PS vs. 200 nm β-Glc-PS	****	*
C. albicans + 200 nm β-Glc-PS vs. 80 nm β-Glc-PS	****	*
C. albicans + 200 nm β-Glc-PS vs. 26 nm β-Glc-PS	****	*
C. albicans + 80 nm β-Glc-PS vs. C. albicans + 26 nm β-Glc-PS	****	ns
C. albicans + 80 nm β-Glc-PS vs. 200 nm β-Glc-PS	***	ns
C. albicans + 80 nm β-Glc-PS vs. 80 nm β-Glc-PS	***	ns
C. albicans + 80 nm β-Glc-PS vs. 26 nm β-Glc-PS	***	ns
C. albicans + 26 nm β-Glc-PS vs. 200 nm β-Glc-PS	****	***
C. albicans + 26 nm β-Glc-PS vs. 80 nm β-Glc-PS	****	***
C. albicans + 26 nm β-Glc-PS vs. 26 nm β-Glc-PS	****	***
200 nm β-Glc-PS vs. 80 nm β-Glc-PS	ns	ns
200 nm β-Glc-PS vs. 26 nm β-Glc-PS	ns	ns
80 nm β-Glc-PS vs. 26 nm β-Glc-PS	ns	ns
Meio vs. 200 nm COOH-PS	****	**
Meio vs. 80 nm COOH-PS	***	**
Meio vs. 26 nm COOH-PS	***	*
PMA vs. 200 nm COOH-PS	***	ns
PMA vs. 80 nm COOH-PS	****	ns
PMA vs. 26 nm COOH-PS	****	ns
C. albicans vs. 200 nm COOH-PS	ns	ns
C. albicans vs. 80 nm COOH-PS	ns	ns
C. albicans vs. 26 nm COOH-PS	ns	ns
200 nm COOH-PS vs. 80 nm COOH-PS	ns	ns
200 nm COOH-PS vs. 26 nm COOH-PS	ns	ns
80 nm COOH-PS vs. 26 nm COOH-PS	ns	ns

Figure S8. Effect of COOH-PS NP treatment in BMDC proinflammatory cytokine production. Quan-

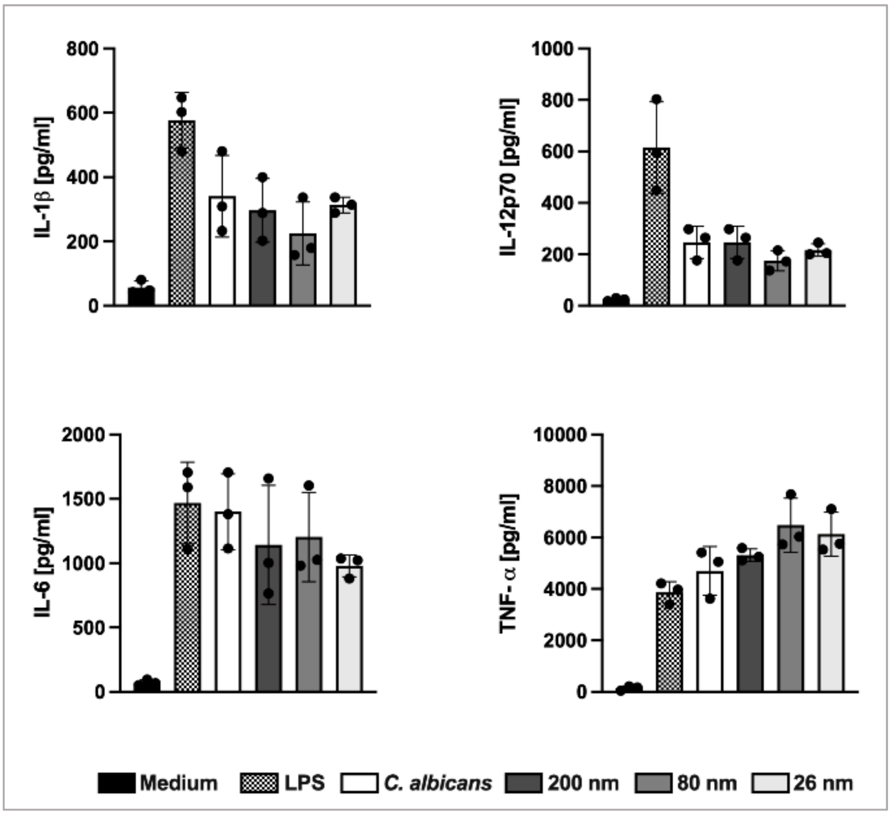

tification by ELISA of TNF-α, IL-1β, IL-6 and IL-12p70 levels, as indicated, in the culture supernatants of BALB/c mice BMDC. BMDC were incubated for 24h with live *C. albicans* at a MOI of 1 DC: 5 yeast cells. 200, 80 and 26 nm COOH-PS NP (10 μg/mL) were added 1h prior to infection. Each condition was set in triplicate. Bars correspond to means ± SD. Data were analyzed using One-way ANOVA with Tukey's post Hoc test.

Table S3. Statistical analysis of BMDC proinflammatory cytokine production. One-way ANOVA with Tukey's post Hoc test (*P<0.05; **P<0.01; ***P<0,001; ****P<0.0001; ns- not significant).

Tukey's multiple comparisons test	*IL-1β*	*IL-12p70*	*IL-6*	*TNF-α*
Medium vs. LPS	****	****	****	****
Medium vs. C. albicans	***	*	****	****
Medium vs. C. albicans + β-Glc	**	*ns*	***	****
Medium vs. C. albicans + 200 nm β-Glc-PS	*ns*	*ns*	*ns*	**
Medium vs. C. albicans + 80 nm β-Glc-PS	*ns*	*ns*	*ns*	*ns*
Medium vs. C. albicans + 26 nm β-Glc-PS	*ns*	*ns*	*ns*	*
Medium vs. 200 nm β-Glc-PS	*ns*	*ns*	*ns*	*ns*
Medium vs. 80 nm β-Glc-PS	*ns*	*ns*	*ns*	*ns*
Medium vs. 26 nm β-Glc-PS	*ns*	*ns*	*ns*	*ns*
LPS vs. C. albicans	**	****	*ns*	*ns*
LPS vs. C. albicans + β-Glc	**	****	*ns*	*ns*
LPS vs. C. albicans + 200 nm β-Glc-PS	****	****	****	****
LPS vs. C. albicans + 80 nm β-Glc-PS	****	****	****	****
LPS vs. C. albicans + 26 nm β-Glc-PS	****	****	***	****
LPS vs. 200 nm β-Glc-PS	****	****	****	****
LPS vs. 80 nm β-Glc-PS	****	****	****	****
LPS vs. 26 nm β-Glc-PS	****	****	****	****
C. albicans vs. C. albicans + β-Glc	*ns*	*ns*	*ns*	*ns*
C. albicans vs. C. albicans + 200 nm β-Glc-PS	*ns*	*ns*	****	****
C. albicans vs. C. albicans + 80 nm β-Glc-PS	*ns*	*ns*	****	****
C. albicans vs. C. albicans + 26 nm β-Glc-PS	*ns*	*ns*	***	****
C. albicans vs. 200 nm β-Glc-PS	*	*	****	****
C. albicans vs. 80 nm β-Glc-PS	*	*	****	****
C. albicans vs. 26 nm β-Glc-PS	*ns*	*	****	****
C. albicans + β-Glc vs. C. albicans + 200 nm β-Glc-PS	*ns*	*ns*	***	****
C. albicans + β-Glc vs. C. albicans + 80 nm β-Glc-PS	*ns*	*ns*	***	****
C. albicans + β-Glc vs. C. albicans + 26 nm β-Glc-PS	*ns*	*ns*	*ns*	****
C. albicans + β-Glc vs. 200 nm β-Glc-PS	*	*ns*	***	****
C. albicans + β-Glc vs. 80 nm β-Glc-PS	*ns*	*ns*	***	****
C. albicans + β-Glc vs. 26 nm β-Glc-PS	*ns*	*ns*	***	****
C. albicans + 200 nm β-Glc-PS vs. C. albicans + 80 nm β-Glc-PS	*ns*	*ns*	*ns*	*ns*
C. albicans + 200 nm β-Glc-PS vs. C. albicans + 26 nm β-Glc-PS	*ns*	*ns*	*ns*	*ns*
C. albicans + 200 nm β-Glc-PS vs. 200 nm β-Glc-PS	*ns*	*ns*	*ns*	**
C. albicans + 200 nm β-Glc-PS vs. 80 nm β-Glc-PS	*ns*	*ns*	*ns*	**
C. albicans + 200 nm β-Glc-PS vs. 26 nm β-Glc-PS	*ns*	*ns*	*ns*	*
C. albicans + 80 nm β-Glc-PS vs. C. albicans + 26 nm β-Glc-PS	*ns*	*ns*	*ns*	*ns*
C. albicans + 80 nm β-Glc-PS vs. 200 nm β-Glc-PS	*ns*	*ns*	*ns*	*ns*
C. albicans + 80 nm β-Glc-PS vs. 80 nm β-Glc-PS	*ns*	*ns*	*ns*	*ns*
C. albicans + 80 nm β-Glc-PS vs. 26 nm β-Glc-PS	*ns*	*ns*	*ns*	*ns*
C. albicans + 26 nm β-Glc-PS vs. 200 nm β-Glc-PS	*ns*	*ns*	*ns*	*
C. albicans + 26 nm β-Glc-PS vs. 80 nm β-Glc-PS	*ns*	*ns*	*ns*	*
C. albicans + 26 nm β-Glc-PS vs. 26 nm β-Glc-PS	*ns*	*ns*	*ns*	*
200 nm β-Glc-PS vs. 80 nm β-Glc-PS	*ns*	*ns*	*ns*	*ns*
200 nm β-Glc-PS vs. 26 nm β-Glc-PS	*ns*	*ns*	*ns*	*ns*
80 nm β-Glc-PS vs. 26 nm β-Glc-PS	*ns*	*ns*	*ns*	*ns*
Medium vs. 200 nm COOH-PS	*	*ns*	*	****
Medium vs. 80 nm COOH-PS	*ns*	*ns*	**	****
Medium vs. 26 nm COOH-PS	*	*ns*	**	****
LPS vs. C. albicans	*	**	*ns*	*ns*
LPS vs. 200 nm COOH-PS	*	**	*ns*	*ns*
LPS vs. 80 nm COOH-PS	**	***	*ns*	**
LPS vs. 26 nm COOH-PS	*	***	*ns*	*
C. albicans vs. 200 nm COOH-PS	*ns*	*ns*	*ns*	*ns*

C. albicans vs. 80 nm COOH-PS	*ns*	*ns*	*ns*	*ns*
C. albicans vs. 26 nm COOH-PS	*ns*	*ns*	*ns*	*ns*
200 nm COOH-PS vs. 80 nm COOH-PS	*ns*	*ns*	*ns*	*ns*
200 nm COOH-PS vs. 26 nm COOH-PS	*ns*	*ns*	*ns*	*ns*
80 nm COOH-PS vs. 26 nm COOH-PS	*ns*	*ns*	*ns*	*ns*
